# Human-to-*Anopheles dirus* mosquito transmission of the anthropozoonotic malaria parasite, *Plasmodium knowlesi*

**DOI:** 10.1186/s13071-024-06500-5

**Published:** 2024-10-04

**Authors:** Chalermpon Kumpitak, Apisak Duangmanee, Waraporn Thongyod, Nattawan Rachaphaew, Chayanut Suansomjit, Khajohnpong Manopwisedjaroen, Pyae Linn Aung, Hisham Ahmed Imad, Liwang Cui, Jetsumon Sattabongkot, Wang Nguitragool, Sirasate Bantuchai

**Affiliations:** 1 Mahidol Vivax Research Unit, Faculty of Tropical Medicine, Mahidol University, Bangkok, 10400 Thailand; 2 Department of Clinical Tropical Medicine, Faculty of Tropical Medicine, Mahidol University, Bangkok, Thailand; 3 Department of Internal Medicine, Morsani College of Medicine, University of South Florida, Tampa, FL USA; 4 Department of Molecular Tropical Medicine and Genetics, Faculty of Tropical Medicine, Mahidol University, Bangkok, Thailand

**Keywords:** Malaria, *Plasmodium knowl*esi, *Anopheles dirus*, Sporozoite, Zoonosis

## Abstract

**Background:**

*Plasmodium knowlesi*, identified as the fifth human malaria parasite, has rapidly spread across various Southeast Asian countries, yet uncertainties persist regarding its human-mosquito-human transmission. Therefore, this study aims to explore the transmission potential of *P. knowlesi* from human blood to mosquitoes.

**Methods:**

A direct membrane-feeding assay was conducted by infecting laboratory-reared female *Anopheles dirus* mosquitoes with *P. knowlesi*-infected human blood from a single patient presenting with febrile malaria. Mosquitoes were dissected 7 days post-infection under a stereomicroscope to detect oocysts in the midgut, stained with mercurochrome. Salivary glands were examined 14 days post-infection for the presence of sporozoites. Malaria diagnosis employed microscopy by expert microscopists and nested PCR assays.

**Results:**

Upon dissecting 745 out of 1439 blood-fed *An. dirus* mosquitoes on day 7 post-infection, two oocysts were identified in the midguts of two mosquitoes (0.27%). An additional 694 mosquitoes were dissected for salivary glands on day 14 post-infection, with three mosquitoes (0.43%) exhibiting sporozoites. Further confirmation by nested-PCR assay verified these sporozoites as belonging to the *P. knowlesi* species.

**Conclusions:**

The findings underscore the potential transmission of *P. knowlesi* from human blood to mosquitoes. The significance of these findings necessitates further investigation, such as repeating similar experiments among natural vectors, to gain deeper insights into the transmission dynamics of *P. knowlesi* in Southeast Asia.

**Graphical Abstract:**

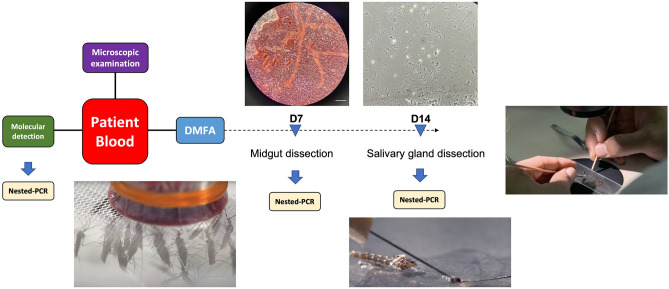

**Supplementary Information:**

The online version contains supplementary material available at 10.1186/s13071-024-06500-5.

## Background

*Plasmodium knowlesi*, a simian malaria parasite, is recognized as one of the contributors to human malaria cases in Southeast Asia [1, 2]. This parasite is prevalent in areas where natural reservoir hosts such as long-tailed (*Macaca fascicularis*) and pig-tailed (*Macaca nemestrina*) macaques inhabit [2, 3]. Those monkeys also serve as natural reservoirs for other simian malaria, including *Plasmodium cynomolgi, P. inui*, *P. fieldi*, *P. coatneyi* and *P. simiovale* [2–8]. The incidence of *P. knowlesi* malaria exhibits geographical variation, with the highest incidence observed in Malaysia [1]. Sporadic cases of *P. knowlesi* have been reported in Thailand since 2004 [3, 4], but recently there has been a rapid increase in the number of reported knowlesi malaria cases, as reported to the Center for Disease Control in Thailand (Additional file 1) [9]. It remains unclear whether this rise is due to the introduction of the parasites or persistently circulating parasites that had been under-detected. While the prevalence of *Plasmodium knowlesi* is considerably lower than that of *P. falciparum* and *P. vivax* [3, 5, 10], this parasite represents an important public health threat to the endemic communities [10]. There have been reports of several cases of co-infections involving *P. knowlesi* and other human malaria species found in Thailand, particularly in the southern regions [3, 5, 11]. Due to the 24-h intraerythrocytic cycle, *P. knowlesi* parasitemia can escalate quickly, leading to severe illness and death if not promptly treated [1, 12, 13]. Hence, even though effective drugs are available, severe and fatal cases of *P. knowlesi* malaria are occasionally observed [14]. The transmission of *P. knowlesi* is considered mostly zoonotic as natural human-to-human transmission has not been demonstrated.

The transmission of *P. knowlesi* is primarily limited to the *Anopheles leucosphyrus* and *An. umbrosus* groups of mosquito vectors found in Southeast Asia [15, 16]. This diverse group of mosquitoes exhibits a multitude of species across the region, each with varying vectorial capacities [16]. Importantly, the *An. leucosphyrus* Group serves as vectors not only for *P. knowlesi* but also for other malaria parasites, including *P. falciparum* and *P. vivax* [17]. Despite the limited number of studies, investigations into suspected jungle transmission sites of *P. knowlesi*, such as those in Malaysian Borneo [15, 18], Kuala Lipis [19], and Khanh Phu (South Vietnam) [20], have been conducted, involving the sampling of mosquitoes for *P. knowlesi* vectors. The identification of specific vectors varies across regions, with *Anopheles balabacensis* implicated in *P. knowlesi* transmission in Sabah and northern Sarawak, Malaysian Borneo [15, 18], and *An. latens*, known to feed on both humans and long-tailed macaques, has been identified as the primary vector in the Kapit Division of Sarawak [18]. Meanwhile, studies in Kuala Lipis reveal *Anopheles cracens*, also within the *leucosphyrus* group, as the predominant malaria vector, with some mosquitoes testing positive for *P. knowlesi* sporozoites or oocysts [19]. This study, conducted in a village setting, with *An. cracens* the only vector in the site, was observed only outdoors, with none of the mosquitoes captured inside human dwellings, decreasing the likelihood of human-mosquito-human transmission of *P. knowlesi* within households [19]. In Southern Vietnam, *Anopheles dirus* has been identified as the predominant malaria vector species. Notably, some *An. dirus* mosquitoes were found to be co-infected with *Plasmodium knowlesi*, *P. falciparum*, *P. vivax* and *P. malariae* [20]. Given the complexity and diversity of the *leucosphyrus* group of mosquitoes in Southeast Asia, extrapolating information between locations is challenging. Therefore, it is important to gather information on the local vectors of *P. knowlesi* within the region. This is essential for identifying areas where the human population is at risk [21] and monitoring changes in vector behavior that may indicate a host switch for *P. knowlesi* from macaque-to-human to human-mosquito-human transmission. It has been reported that sporozoites of *P. knowlesi* were found in the salivary glands after experimental infection with *Anopheles stephensi*, *An. vagus*, *An. maculatus* (identified as a vector in Thailand), *An. kochi* and *An. quadrimaculatus* [22].

Notably, *An. stephensi* was initially utilized to transmit the parasite from monkey to monkey [23]; however, sporozoite counts in the salivary glands were relatively low. In experimental transmission studies, Chin et al. [24] employed laboratory-reared *An. balabacensis* mosquitoes to transmit *P. knowlesi* to both human volunteers and monkeys. Then, Sullivan et al. [25] transmitted three different strains of *P. knowlesi* to New World monkeys using *An. dirus* mosquitoes. Rosenberg [26] determined that *Anopheles freeborni* mosquitoes were unable to transmit the parasite because sporozoites could not invade the salivary glands. Although several studies have conducted experimental transmission in vectors, direct evidence showing the possibility of *P. knowlesi* parasite transmitting from human to human is still lacking. In this study, to explore the transmission potential of *P. knowlesi* in human blood to mosquitoes, we conducted direct membrane feeding assay (DMFA) using *P. knowlesi*-infected blood from a human malaria patient with laboratory-reared *An. dirus* mosquitoes.

## Methods

### Blood sample collection

**Fig. 1:**
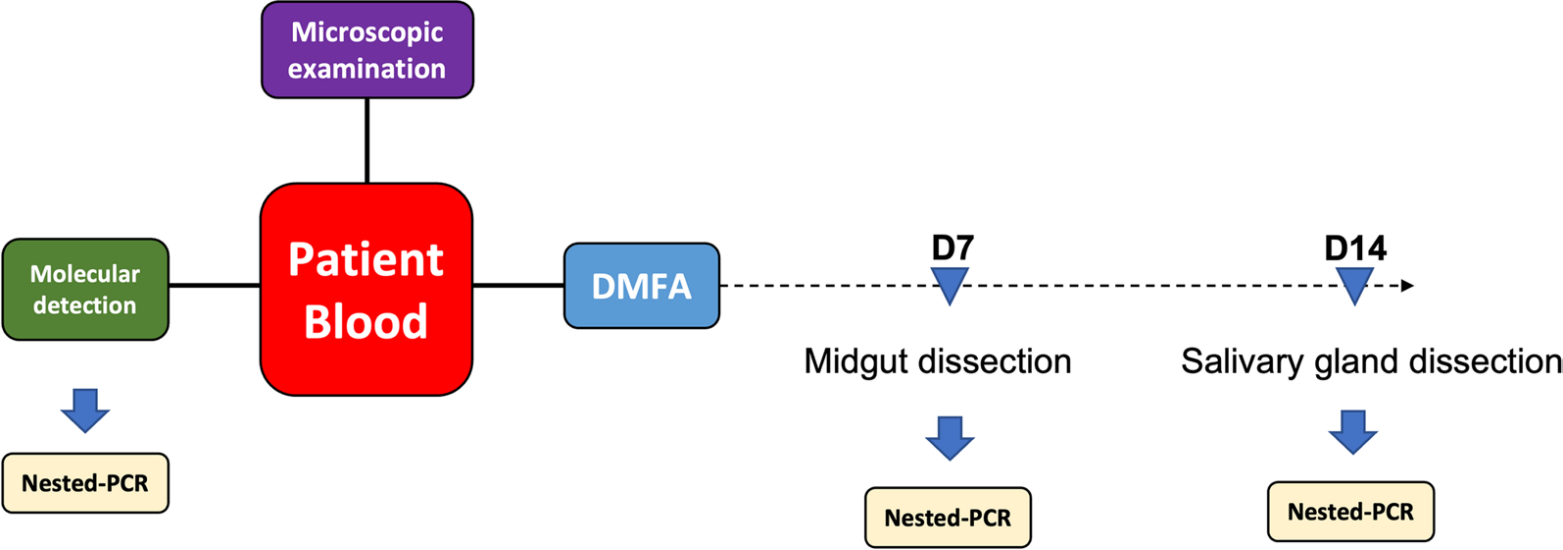
Workflow for blood sample processing and mosquito feeding

In early February 2023, we recruited a malaria-positive patient who presented at the malaria clinic in Yala province, Southern Thailand, to participate in this study. Venous blood was collected in heparin-containing vacutainer blood collection tubes, and blood spots on filter paper were also prepared. To maintain the sample in optimal conditions, the collected blood was kept in a temperature-controlled container at 36–38 °C during transport from the malaria clinic to the laboratory in Bannang Sata District, Yala Province. The blood sample was then prepared for mosquito feeding, as shown in the flowchart (Fig. 1).

### Direct membrane feeding assay (DMFA) and mosquito dissection

We conducted the DMFA using *An. dirus* mosquitoes as previously described [27]. Briefly, a membrane feeding apparatus was set up at the field laboratory, consisting of four large plastic mosquito containers (mosquito carton) with glass feeders, each covered with a Baudruche membrane. The glass feeders were interconnected with rubber tubing attached to the water bath circulator, which maintained the temperature at around 37–38 °C.

Eight milliliters of whole blood were centrifuged to separate packed red blood cells (RBCs) and plasma. The plasma was removed and collected separately. The separated packed RBCs were washed with incomplete RPMI 1640 medium (Thermo-Fisher). Next, human AB naïve serum (The Thai Red Cross Society) was added to packed RBCs to achieve the final suspension volume of 8 ml. Four glass feeders were used for a mosquito carton containing 400 mosquitoes; each glass feeder was filled with 500 µl blood suspension (total 4 glass feeders/carton).

After having been starved of sugar for about 6 h, a total of 1600 5- to 7-day old female *An. dirus* mosquitoes (400 mosquitoes/carton) were allowed to feed on the parasitized blood (2 ml/carton) through the membrane for 30 min. Unfed and partially fed mosquitoes were removed, retaining only the fully engorged ones. Mosquitoes were dissected under a stereomicroscope on day 7 post-infection to examine the presence of oocysts from mosquito midgut staining with mercurochrome. The presence of sporozoites in the mosquito salivary glands was examined on day 14 post-infection.

### Malaria diagnosis

#### Detection of blood stage malaria parasites through blood smear examination

The thick and thin blood smear slides were stained using 10% Giemsa and examined by an expert microscopist under a light microscope at 1000 × magnification. The number of parasites per 500 leukocytes was counted on the thick smear to estimate parasitemia. Parasitemia was expressed as the percentage of infected RBCs. The thin smear was used to identify the malaria parasite species.

#### DNA extraction and nested PCR for *Plasmodium* species identification

Genomic DNA was extracted from whole blood using QIAmp DNA Blood Mini Kit (Qiagen, Germany) according to the manufacturer’s instructions. Nested PCR targeting the 18S rRNA genes was performed as previously described [11]. The resulting PCR products were visualized by electrophoresis using a 1.5% agarose gel containing GelRed nucleic acid stain (Sigma-Aldrich). After dissection, mosquito midguts and salivary glands were also subjected to 18S rRNA nested PCR to confirm the infection.

## Results

**Fig. 2:**
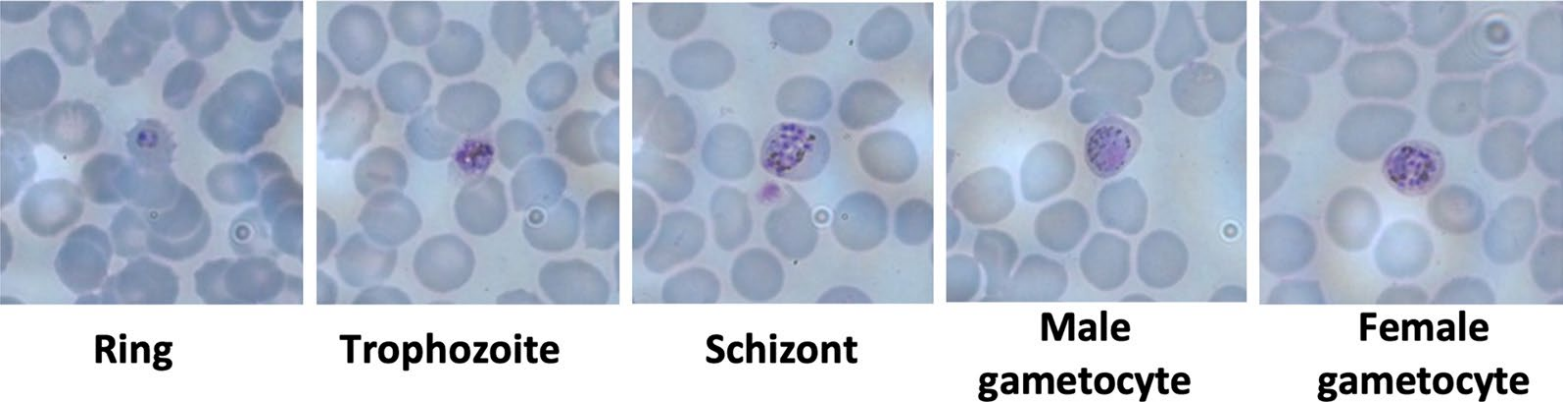
Giemsa-stained thin blood smear images from patient’s blood of each parasite stage: ring, trophozoite, schizont, male gametocyte and female gametocyte observed at 100× objective magnification using light microscope

A malaria case presenting at a clinic in Yala Province was suspected to be infected with *P. vivax* after microscopic examination by local malaria clinic staff. The blood smear was re-examined at the Mahidol Vivax Research Unit (MVRU). The parasites from thick-smear slides were counted and differentiated as: 34 ring stages, 170 schizonts, 1 male gametocyte, and 5 female gametocytes. The parasite morphology at each stage, without red cell enlargement, was consistent with *P. knowlesi* infection (Fig. 2). Nested PCR targeting the 18S rRNA genes confirmed the presence of *P. knowlesi* with a specific PCR product.

A DMFA was performed to determine the vector competence of *An. dirus* for *P. knowlesi* parasites in infected blood samples. A total of 1600 female *An. dirus* mosquitoes (4 cartons, 400 mosquitoes/carton) were fed with 8 ml *P. knowlesi*-infected blood (2 ml/carton) for 30 min. After removing 161 unengorged mosquitoes, 1439 mosquitoes were retained for dissection (Table 1).

**Table 1:** Engorgement rates of *Anopheles dirus* mosquitoes

Mosquito carton no.	Mosquito spp.	Engorged mosquito(s)	Total mosquito(s)
1	*An. dirus*	384 (96.0%)	400
2	*An. dirus*	370 (92.5%)	400
3	*An. dirus*	331 (82.8%)	400
4	*An. dirus*	354 (88.5%)	400

spp., species; *An. dirus*, *Anopheles dirus*

**Fig. 3:**
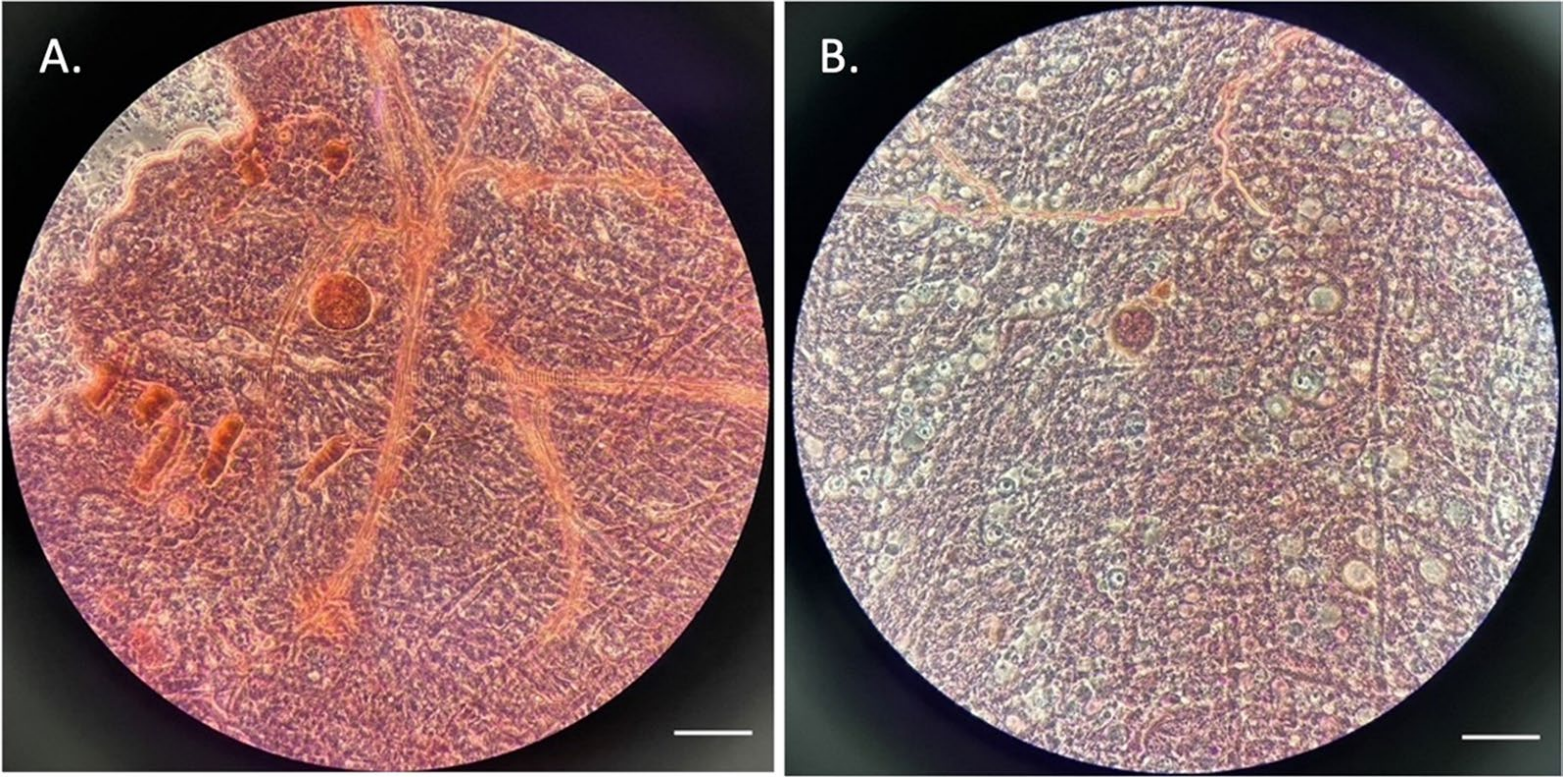
Oocyst-positive mosquito midguts. A and B: *Anopheles dirus* midgut containing an oocyst from each individual mosquito from light microscope at 40· objective magnification with mercurochrome staining. Scale bar 1 µm

**Fig. 4:**
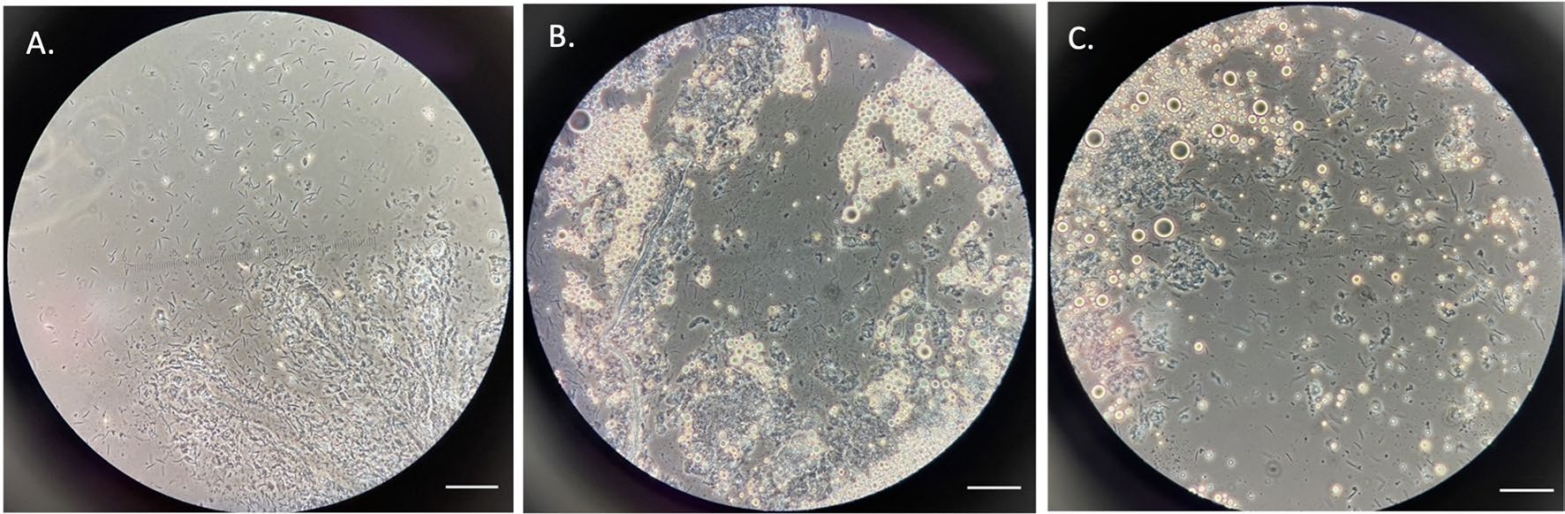
Mosquito salivary glands containing sporozoites. A, B and C: *Anopheles dirus* salivary glands containing sporozoites from each individual mosquito from phase contrast microscope at 40× objective magnification without staining. Scale bar, 1 µm

**Fig. 5:**
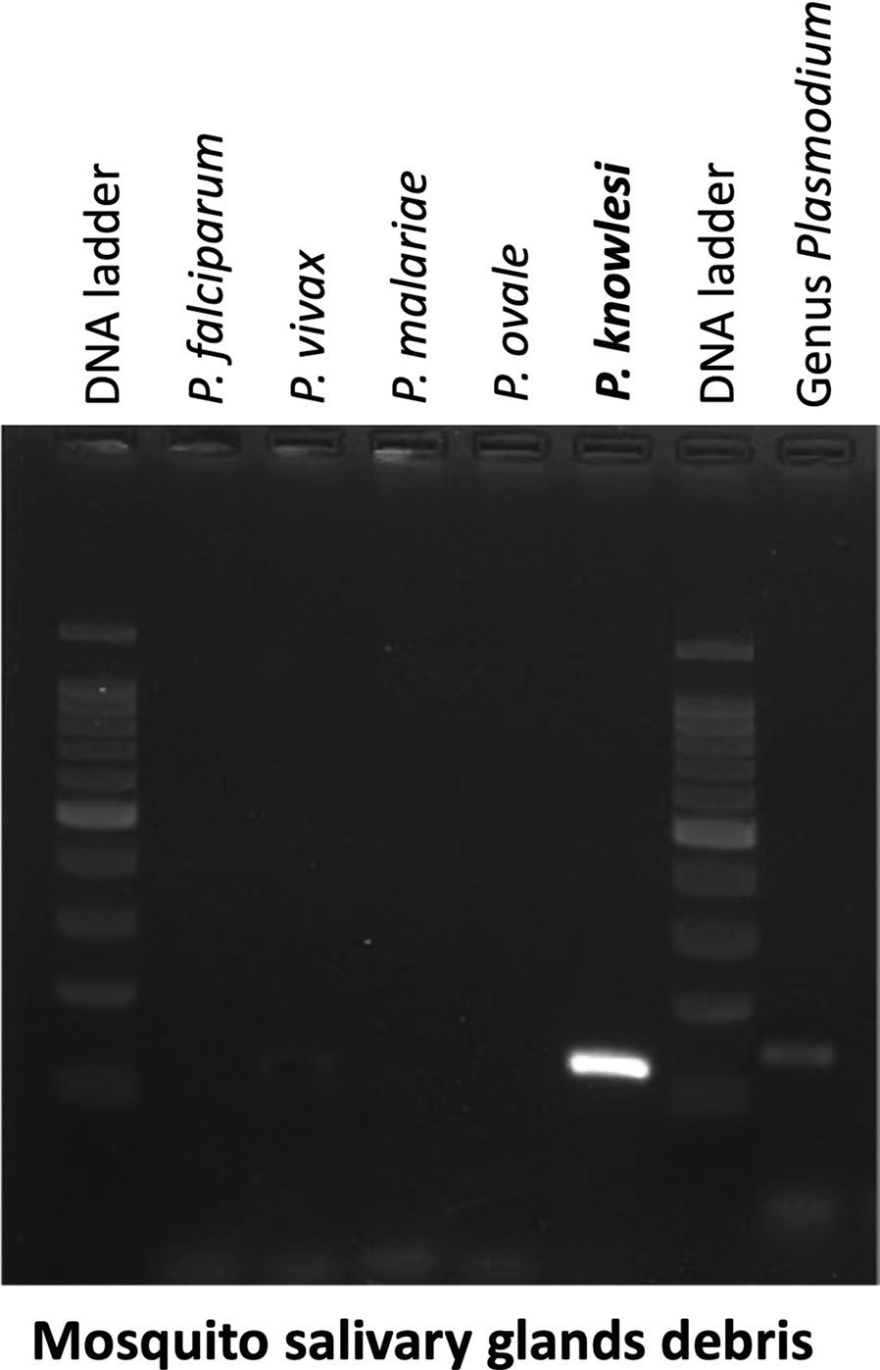
Nested-PCR gel result for *Plasmodium* spp. detection from mosquito salivary glands debris. Lane 1: DNA ladder, Lane 2: *Plasmodium falciparum*, Lane 3: *P. vivax,* Lane 4: *P. malariae*, Lane 5: *P. ovale,* Lane 6: *P. knowlesi*, Lane 7: DNA ladder and Lane 8: Genus *Plasmodium*

On day 7 post-infection, 745 mosquitoes were dissected to screen for midgut oocysts (Fig. 3A, B). From these, two oocysts were found in two mosquitoes, reflecting a 0.27% infection rate. On day 14, a total of 694 mosquitoes were examined for sporozoite infections, and three were found to be positive, with 700, 397, and 540 sporozoites in their salivary glands (Fig. 4A, B, C). All sporozoite-positive salivary glands from the three mosquito carcasses were pooled and used for DNA extraction and nested PCR (Fig. 5) analysis, which confirmed *P. knowlesi* species.

## Discussion

The malaria trend in Thailand has demonstrated a substantial decline over the past decade, with a noteworthy reduction of almost 90% in total cases by 2021 compared to 2011 [10]. However, a resurgence of malaria cases has been observed since 2022, particularly among mobile and migrant populations residing in provinces bordering Myanmar [9]. This resurgence is further complicated by sporadic cases of *P. knowlesi* reported across Thailand since 2004, primarily originating from Southern provinces [3, 4]. Initially presumed to be imported cases from neighboring Malaysia, where *P. knowlesi* is the predominant human malaria species [28], the subsequent increase in reported cases suggests a shift towards localized transmission. This shift is likely facilitated by the presence of macaques and mosquito vectors of the *Leucosphyrus* group [29, 30]. Moreover, the increase in *P. knowlesi* infections is inversely proportional to the decline in the incidence of other species. A similar pattern was observed in Sabang, Indonesia, where *P. knowlesi* emerged following the elimination of *P. falciparum* and *P. vivax* [31, 32]. Another possibility is that when the human host is occupied by an adapted *Plasmodium* species, it becomes less susceptible to *P. knowlesi*. Specifically, the removal of *P. falciparum* may create a more permissive environment for *P. knowlesi* [32].

A recent study has corroborated the historical presence of *P. knowlesi* in Thailand, as evidenced by samples collected during 1990s, providing additional support for the understanding of the historical epidemiology of this malaria species in the region [4]. Accordingly, data from the national malaria elimination dashboard reveal a dramatic surge in *P. knowlesi* cases, escalating from a single case in 2016 to almost 300 cases by late 2023, spanning 21 provinces in the country [9]. Despite an extensive review of the available literature and application of modeling approaches to malaria data, there is no evidence to support the occurrence of human-to-human transmission of *P. knowlesi* via mosquitoes [33, 34]; the increasing trend and rapid geographical spread underscore the epidemiology of knowlesi malaria, emphasizing the need for tailored interventions to control disease transmission. In this study, laboratory-reared *An. dirus* has been identified as a potential vector for human-mosquito-human *P. knowlesi* transmission. This conclusion is drawn from membrane-feeding experiments using infected blood containing *P. knowlesi* parasites, despite the relatively small number of infected mosquitoes. Further research is warranted to continuously monitor potential transmission routes beyond the conventional sylvatic cycle.

The anopheline mosquitoes belonging to the *Leucosphyrus* group, including *Anopheles dirus*, *An. latens* and *An. balabacensis*, are recognized as primary vectors proficient in supporting the complete sporogonic cycle of *P. knowlesi* parasites in Thailand and other Southeast Asian countries [20, 30, 35, 36]. These vectors can transmit other simian malaria parasites besides major human malaria [17]. Among these species, alongside *Anopheles maculatus* and *An. minimus*, *An. dirus* stands out as a major vector species responsible for transmitting most malaria parasite species in Thailand, exhibiting a widespread distribution throughout the country [35]. Several distinctive characteristics of *An. dirus* relevant to *P. knowlesi* include their preferential habitat in forest-related locations, ecologically independent of human activities and other environmental factors [37]. Their dual propensity for both anthropophilic and zoophilic behavior may contribute to the escalating transmission of *P. knowlesi* malaria [38–40]. Furthermore, certain investigations have identified the presence of sporozoites or DNA of *P. knowlesi* parasites in the salivary glands of *An. dirus* [17, 20]. This study employed a laboratory-reared mosquito colony of *An. dirus* that has been successfully utilized in numerous studies to validate its transmissibility and competence [41]. In addition, similar experiments with other mosquitoes within the *Leucosphyrus* Group are needed for a comprehensive understanding of their potential roles in disease transmission.

The malaria transmission from humans to *Anopheles* mosquitoes represents a critical bottleneck in the context of *P. knowlesi* transmission, necessitating monitoring to comprehend its rapid dissemination and formulate effective control measures. Moreover, *P. knowlesi* exhibits a distinct asexual life cycle, notably shorter than in other major malaria species, resulting in a rapid increment of parasitemia within 24 h [28, 42]. This characteristic underscores the need for further and continuous investigation. To this end, membrane-feeding experiments conducted under laboratory conditions are indispensable, although experiments involving natural vectors remain essential [30]. The efficacy of such experiments has been well demonstrated in for *P. falciparum* and *P. vivax* [43]. *Anopheles dirus* has been extensively employed in numerous experiments to delineate their asexual life cycles, involving feeding them *P. knowlesi*-infected blood from macaques as well as in vitro-cultivated *P. knowlesi* H strain [43, 44]. While some studies have reported the successful formation of oocysts and sporozoites within 5–10 days, other experimental infection studies yielded negative results, failing to document parasite development [45, 46]. Nevertheless, membrane-feeding assays utilizing infected human blood are limited but essential to identify and enhance understanding of the potential human-mosquito-human transmission pathway [17]. The present study addresses this gap, yielding results that underscore the potential of human-to-mosquito transmission and demonstrate a need for future studies incorporating natural vectors, where feasible.

This study has notable strengths and limitations. One key limitation is that it involved blood samples from a single patient infected with *P. knowlesi*. Future studies should aim to recruit a larger number of patients from diverse regions, capturing a broader range of parasite densities, including varying gametocyte levels. This would allow for more comprehensive insights into parasite transmission dynamics. The MVRU currently maintains colonies of *An. dirus* and *An. minimus*. In this study, we used *An. dirus* colonies for DMFA, contributing to a better understanding of vector competence for *P. knowlesi*. However, given the geographical variation in the distribution of competent malaria vectors, as well as differences in transmission rates and parasite development across regions [47], further DMFA experiments using other vector species, such as *An. minimus* or other potential vectors of *P. knowlesi*, are essential for future research. This would provide a more comprehensive understanding of *P. knowlesi* transmission dynamics across different settings.

## Conclusions

In summary, the detection of sporozoite formation in the salivary glands of three mosquitoes implies the potential transmission of *P. knowlesi* from humans to *Anopheles* mosquitoes. This result emphasizes the conceivable transmission of *P. knowlesi* from human blood to mosquitoes. The significance of these findings warrants further exploration, such as repeating similar experiments among natural vectors, to gain deeper insights into the transmission dynamics of *P. knowlesi* in Southeast Asia.

## Supplementary Information

Supplementary material 1. Malaria case report in Thailand (2012–2023). Green circles represent the annual number of *Plasmodium falciparum* cases, blue triangles represent *P. vivax* cases, and red diamonds represent *P. knowlesi* cases from 2012 to 2023. The left vertical axis corresponds to *P. falciparum* and *P. vivax*; the right vertical axis corresponds to *P. knowlesi*. Parasite confirmation was conducted by microscopy.

## Data Availability

No datasets were generated or analysed during the current study.

## Abbreviations

DMFA: Direct membrane feeding assay
RBCs: Red blood cells
PCR: Polymerase chain reaction
DNA: Deoxyribonucleic acid
RNA: Ribonucleic acid
MVRU: Mahidol Vivax Research Unit
