# Correction: Human-to-*Anopheles dirus* mosquito transmission of the anthropozoonotic malaria parasite, *Plasmodium knowlesi*

**DOI:** 10.1186/s13071-025-07061-x

**Published:** 2025-09-29

**Authors:** Chalermpon Kumpitak, Apisak Duangmanee, Waraporn Thongyod, Nattawan Rachaphaew, Chayanut Suansomjit, Khajohnpong Manopwisedjaroen, Pyae Linn Aung, Hisham Ahmed Imad, Liwang Cui, Jetsumon Sattabongkot, Wang Nguitragool, Sirasate Bantuchai

**Affiliations:** 1 Mahidol Vivax Research Unit, Faculty of Tropical Medicine, Mahidol University, Bangkok, 10400 Thailand; 2 Department of Clinical Tropical Medicine, Faculty of Tropical Medicine, Mahidol University, Bangkok, Thailand; 3 Department of Internal Medicine, Morsani College of Medicine, University of South Florida, Tampa, FL USA; 4 Department of Molecular Tropical Medicine and Genetics, Faculty of Tropical Medicine, Mahidol University, Bangkok, Thailand

**Correction: Parasites & Vectors (2024) 17:415** 10.1186/s13071-024-06500-5

Following publication of the article, the EC approval number in the declaration ‘Ethics approval and consent to participate’ has been corrected (to MUTM 2018-016-08). The authors thank you for reading this erratum [1].

## References

[1] Kumpitak C, Duangmanee A, Thongyod W, et al. Human-to-*Anopheles dirus* mosquito transmission of the anthropozoonotic malaria parasite, *Plasmodium knowlesi*. Parasit Vectors. 2024;17:415. 10.1186/s13071-024-06500-5. 39367453 10.1186/s13071-024-06500-5 PMC11451161

