# Supplementary figures and images for: Human-to-Anopheles dirus mosquito transmission of the anthropozoonotic malaria parasite, Plasmodium knowlesi
Source: Parasit Vectors. 2024 Oct 4;17:415. doi: 10.1186/s13071-024-06500-5 (PMC11451161; doi:10.1186/s13071-024-06500-5)

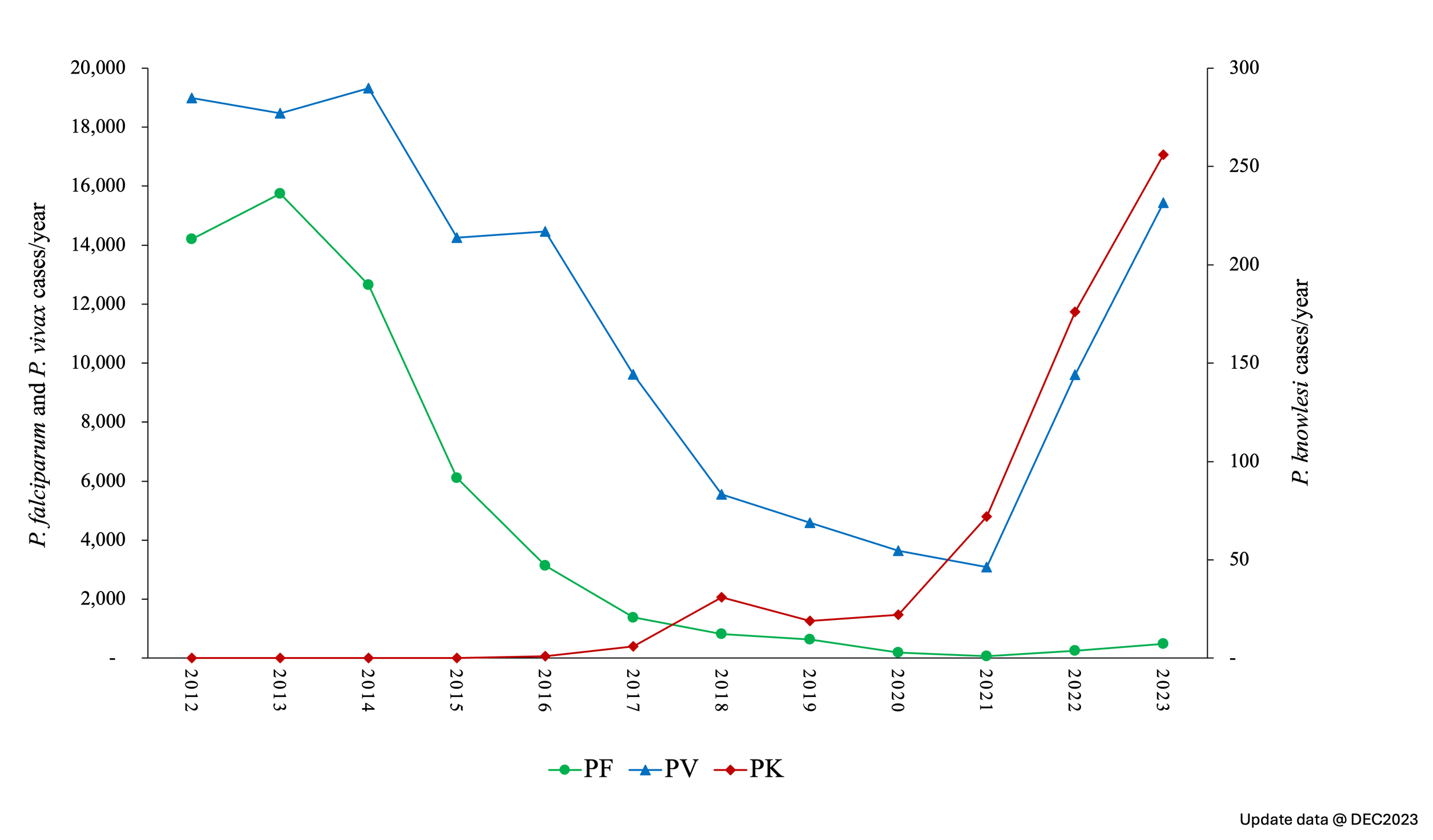

Supplement: Supplementary file 1 — Supplementary material 1. Malaria case report in Thailand (2012–2023). Green circles represent the annual number of Plasmodium falciparum cases, blue triangles represent P. vivax cases, and red diamonds represent P. knowlesi cases from 2012 to 2023. The left vertical axis corresponds to P. falciparum and P. vivax; the right vertical axis corresponds to P. knowlesi. Parasite confirmation was conducted by microscopy. [file 13071_2024_6500_MOESM1_ESM.tif]
